# A Well-Posed Fractional Order Cholera Model with Saturated Incidence Rate

**DOI:** 10.3390/e25020360

**Published:** 2023-02-15

**Authors:** Isa Abdullahi Baba, Usa Wannasingha Humphries, Fathalla A. Rihan

**Affiliations:** 1 Department of Mathematics, Bayero University, Kano 700241, Nigeria; 2 Department of Mathematics, Faculty of Science, King Mongkuts University of Science and Technology Thonburi (KMUTT), Bangkok 10140, Thailand; 3 Department of Mathematical Sciences, College of Science, United Arab Emirates University, Al Ain 15551, United Arab Emirates; 4 Department of Mathematics, Faculty of Science, Helwan University, Cairo 11795, Egypt

**Keywords:** mathematical model, fractional order, Caputo, cholera, well-posedness, saturated incidence rate

## Abstract

A fractional-order cholera model in the Caputo sense is constructed. The model is an extension of the Susceptible–Infected–Recovered (SIR) epidemic model. The transmission dynamics of the disease are studied by incorporating the saturated incidence rate into the model. This is particularly important since assuming that the increase in incidence for a large number of infected individualsis equivalent to a small number of infected individualsdoes not make much sense. The positivity, boundedness, existence, and uniqueness of the solution of the model are also studied. Equilibrium solutions are computed, and their stability analyses are shown to depend on a threshold quantity, the basic reproduction ratio ($R_{0}$). It is clearly shown that if $R_{0}<1,$ the disease-free equilibrium is locally asymptotically stable, whereas if $R_{0}>1$, the endemic equilibrium exists and is locally asymptotically stable. Numerical simulations are carried out to support the analytic results and to show the significance of the fractional order from the biological point of view. Furthermore, the significance of awareness is studied in the numerical section.

## 1. Introduction

Cholera is a prolific diarrheal disease that leads to death in a short period of time if treatment measures are not taken. The disease is estimated to cause about 21,000 to 143,000 deaths from the 1,300,000 to 4,000,000 cholera cases annually. This represents about 1.62% to 3.58% of the reported cases [1]. People who live in slums and refugee camps due to natural disasters, social conflicts, climate change, and economic meltdowns are the most affected. The camps often possess poor drinking water, which serves as a cholera-causing factor [2]. Many of the symptoms of cholera include vomiting, profuse rice–water stool, sunken eyes, cramps, shock, and severe dehydration. *Vibrio cholerae* carriers are those people that are exposed to an incomplete cholera-causing dose, therefore, the disease may not manifest any symptoms in their body [3]. Acute cholera leads to death in a short period that varies from hours up to three days. Exposed individuals have only half a chance of being infected with the disease if the concentration of *Vibrio cholerae* is 105 cells per milliliter. A minimum of 1 liter per day is the least daily consumption of untreated water that may cause the disease [4].

Up to 75% of *Vibrio cholerae* carriers do not show any symptoms of the disease, potentially spreading it through their feces [5]. This causes a big risk of cholera disease and outbreaks. The cholera infection falls into one of three classes: asymptomatic, mild, or severe. In a population of infected individuals, 80% have mild or moderate symptoms and only 20% develop severe watery diarrhea [6].

Mathematical models help in studying the dynamics of a given infectious disease [7,8,9]. The investigation of cholera models using the SIR epidemic model introduced in 1927 [10] provides a cavernous understanding of the transmission mechanisms of the disease, while other models use time-dependent coefficients [11]. This is why many researchers put more effort into constructing and analyzing mathematical models of a cholera epidemic. Cholera dynamical models involve the study of the disease transmission among humans and V. cholerae concentration in contaminated water. Most of the time, the direct and indirect pathway transmission of cholera gives rise to a basic framework for investigating the dynamics of the disease [12,13,14,15,16,17,18].

Due to their hereditary properties and memory description abilities, many researchers prefer to use fractional order derivatives and integrals as tools in the study of mathematical modeling. Nowadays, a fractional differential equation is used to study biological phenomenaby developing related mathematical models [19,20]. This is due to the fact that fractional calculus can be used to explain the retention and heritage properties of many materials more accurately compared to its corresponding integer-order analog [21,22]. In this paper, we use the Caputo fractional order to model the dynamics of cholera in a homogeneous setting.

The mechanism of transmission of any transmissible disease is controlled by a certain function that depends on the subpopulations of infected individuals called the incidence rate. In epidemic models, the most frequently used incidence rates are the standard incidence rate $\frac{\beta SI}{N}$ and the bilinear incidence rate βSI, where β is the contact rate, *S* stands for the susceptible population, *I* stands for the infected population, and *N* is the total population. Assuming the increase in incidence for a large number of infected individualsis equivalent to a small number of infected individuals does not make much sense, hence, there is a need for a more realistic incidence rate of the form g(I)S [23] where g tends to a saturation level, as it is a nonnegative function, such that g(0)=0. To incorporate the effect of the behavioral changes of the susceptible individuals, a more general incidence rate of the form $\frac{\beta SI^{m}}{1+\delta I^{n}}$, where m and n are positive constants and δ is a nonnegative term, was proposed [24]. These types of incidence rates that allow for the possibility of the introduction of psychological effects are called saturated incidence rates; δ and $1+\delta I^{n}$ determine the amount of psychological effect and the inhibition effect, respectively. As the number of infective individuals increases, the rate of infection spread may decrease due to public awareness, potentially leading to contact reduction [25].

Many mathematical models of cholera transmission exist in the literature, and they study the dynamics of the disease. For example, Leo [26] developed an ML reference cholera model that can be used to overcome the existing complexities of modeling the disease. His results indicate, at an average of 87%, that the developed measures can integrate a large number of datasets, including environmental factors, for the timely prediction of cholera epidemics in Tanzania. Daudel et al. [27] constructed and studied a compartmental malaria model. Their results show that the higher implementation of strategies combining awareness programs and therapeutic treatments increases the efficacy of control measures. In [28], a stochastic norovirus epidemic model with a time delay and random perturbations was explored. In [29], a mathematical model for cholera considering vaccination effects was proposed. In [30], Capasso and Paveri-Fontana suggested a mathematical model for the 1973 cholera epidemic in the European Mediterranean region. In 2017, the transmission dynamics of cholera in Yemen were investigated by Nishiura et al. [31]. Lastly, a model containing optimal intervention strategies for cholera control was formulated in [32].

Luchko and Yamamoto [33] proposed a new differential operator with a general kernel function. Due to the existence of flexibility in choosing the kernel, they provide a basis for a broad range of applications [34]. Changing the kernel in the general derivative leads to the discovery of various asymptotic behaviors. Hence, the hidden features of real-world systems are more accurately observed than in the classical sense. However, both properties and applications regarding this operator must be studied in practical situations. There is also the need to state and prove some theorems in order to study models using this operator.

For cholera, the saturated incidence rate is more reasonable than the bilinear incidence rate. This is because it includes the behavioral change and crowding effect of the cholera-infective individuals and prevents the unboundedness of the contact rate by choosing suitable parameters. Motivated by the above discussions, we construct a novel mathematical model that studies the transmission dynamics of cholera in the Caputosense. The model is novel because, to the best of our knowledge, no previous study has analyzed a mathematical model with a saturated incidence rate and fractional derivative for the cholera disease in detail. The main contributions of this research and the new achievements obtained within this manuscript are summarized as follows:

- This paper addresses a new mathematical model of cholera disease which involves the Caputo fractional derivative.
- The fundamental characteristics of the new model are discussed in detail.
- A numerical scheme is developed to carry out numerical simulations.
- The effect of awareness is studied.
- Comparative results in this research show an obvious linkage between the mathematical and biological mechanisms.

Hence, in this paper, we study a fractional-order cholera model with a saturated incidence rate. The main contribution of the paper is the introduction of a more reasonable incidence rate of the form $\frac{\beta SI^{m}}{1+\delta I^{n}}$, which makes more sense when considering that assuming the increase in incidence for a large number of infected individualsis equivalent to a small number of infected individualsdoes not make much sense. It is also the aim of the paper to consider the effect of awareness in the control of cholera and study all the properties of well-posedness.

The paper is arranged as follows: Section 1 gives an introduction, Section 2 gives important definitions and preliminaries, Section 3 gives model formulation, Section 4 gives the well-posedness properties of the model, and Section 5 gives the numerical simulation results to support the analytic results. In Section 6, a discussion and conclusion are given.

## 2. Preliminary Definitions and Theorems

**Definition** **1** [35]**.** *The Caputo fractional derivative of order* α∈(n−1, n] *of* f(x) *is defined as*,

DaCxαf(x)=1Γ(n−α)∫ax(x−t)n−α−1fn(t)dt,       n=[α]+1. 

**Definition** **2** [36]**.** *The linearity of the fractional derivative*.

Let f, g be continuous and b, c be scalars, then

DaRLxα[bf(x)+dg(x)]=bDaRLxαf(x)+dDaRLxαg(x),

DaCxα[bf(x)+dg(x)]=bDaCxαf(x)+dDaCxαg(x).

**Definition** **3** [37]**.** *Contraction*.

An operator f:X →X that maps a metric space onto itself is said to be contractive if for 0<q<1.

d(f(x), f(y))=qd(x, y), ∀ x, y∈X.

**Theorem** **1** [37]**.** *Picard–Banach fixed point*.

Any contractive operator that maps a metric space onto itself has a unique fixed point. Furthermore, if f:X →X is a contractive operator that maps a metric space onto itself and a is its fixed point: f(a)=a; then for any iterative sequence

$$x_{0},\;x_{1}=f\left(x_{0}\right),\;x_{2}=f\left(x_{1}\right),\;\ldots ,\;x_{n+1}=f\left(x_{n}\right),\;\ldots$$

converges to a.

In other words, a is a solution or equilibrium for a continuous dynamical system and a fixed point for a discrete dynamical system.

**Theorem** **2** [36]**.** *The equilibrium solutions* $x^{*}$ *of a system of fractional order differential equation is locally asymptotically stable if all the eigenvalues* $\lambda_{i}$ *of the Jacobian matrix* $\frac{\partial f}{\partial x_{i}}$ *evaluated at the equilibrium points satisfy*

$$\left|\arg\left(\lambda_{i}\right)\right|>\frac{\alpha \pi }{2},\;0<\alpha <1.$$

**Theorem** **3** [36]**.** *Let* $x\left(t\right)\in {\mathbb{R}}^{+}$ *be a continuous and derivable function. Then, for any time instant* $t\geq t_{0}$ *and* α∈(0, 1)

D0Ctα[x(t)−x∗−x∗ln(x(t)x∗)]≤(1−x(t)x∗)D0Ctαx(t), x∗∈ℝ+.

## 3. Model Formulation

The model consists of the following classes: susceptible *S*, latently infected individuals E, infectious individuals I, and those that recovered from the infection R. We also define N(t)=S(t)+E(t)+I(t)+R(t). Assuming homogeneous mixing of the population, the model is given as:

(1) D0CtαS(t)=Λα−βα(1−kα)S(t)I(t)f(I)+γαE(t)+ξαI(t)−μαS(t), D0CtαE(t)=pβα(1−kα)S(t)I(t)f(I)−(γα+ηα+ϕα+μα)E(t),D0CtαI(t)=(1−p)βα(1−kα)S(t)I(t)f(I)+ηαE(t)−(ξα+δα+qα+μα)I(t),D0CtαR(t)=δαI(t)−μαR(t),

subject to the initial conditions;

S(0)>0, E(0)≥0, I(0)≥0, R(0)≥0.

where the function f(I) is given as;

$$f\left(I\right)=1+I^{2}.$$

We then modify the fractional operator via an auxiliary parameter Υ>0 to avoid dimensional mismatching [38] to obtain

(2) Υα−1D0CtαS(t)=Λα−βα(1−kα)S(t)I(t)f(I)+γαE(t)+ξαI(t)−μαS(t),Υα−1D0CtαE(t)=pβα(1−kα)S(t)I(t)f(I)−(γα+ηα+ϕα+μα)E(t), Υα−1D0CtαI(t)=(1−p)βα(1−kα)S(t)I(t)f(I)+ηαE(t)−(ξα+δα+qα+μα)I(t),Υα−1D0CtαR(t)=δαI(t)−μαR(t), 

subject to the initial conditions;

S(0)>0, E(0)≥0, I(0)≥0, R(0)≥0.

The meaning of the parameters in the model are given in Table 1 as follows.

## 4. Well-Posednessof the Model

In this section, the mathematical properties of the model are explored. This consists of the positivity and boundedness, the existence and uniqueness, the computation of equilibrium solutions and basic reproduction ratio, and the stability analysis of the solutions.

### 4.1. Positivity and Boundedness

The positivity of solutions means that the population thrives, while boundedness means that the population growth is restricted naturally due to limited resources.

To show positivity, consider Equation (1),

D0CtαS(t)|S=0=Λα+γαE(t)+ξαI(t)>0, D0CtαE(t)|E=0=pβα(1−kα)S(t)I(t)f(I)≥0, D0CtαI(t)|I=0=ηαE(t)≥0, D0CtαR(t)|R=0=δαI(t)≥0.

Hence, we can observe that the solution of (1) is non-negative.

For the boundedness, observe that,

N(t)=S(t)+E(t)+I(t)+R(t).

Then, by Definition 3, we have

D0CtαN(t)=D0CtαS(t)+D0CtαE(t)+D0CtαI(t)+D0CtαR(t).

Then,

D0CtαN(t)=Λα−μαN(t)−(ϕαE(t)+qαI(t)),D0CtαN(t)≤Λα−μαN(t).

We apply the Laplace transform method to solve the Gronwall’s like inequality with initial condition $N\left(t_{0}\right)\geq 0$ to find

L{D0CFtαN(t)+μαN}≤L{Λα}.

By the linearity of the Laplace transform, we obtain

L{D0CFtαN(t)}+μαL{N(t)}≤L{Λα},

we find,

$$S^{\alpha }L\left\{N\left(t\right)\right\}-\overset{n-1}{\underset{k=0}{\sum }}S^{\alpha -k-1}N^{k}\left(t_{0}\right)+\mu^{\alpha }L\left\{N\left(t\right)\right\}\leq \frac{\Lambda^{\alpha }}{S}.$$

Simplifying, we obtain

$$L\left\{N\left(t\right)\right\}\leq \Lambda^{\alpha }\left(\frac{1}{S}-\frac{1}{S}\frac{1}{\left(1+\frac{\mu^{\alpha }}{S^{\alpha }}\right)}\right)+\overset{n-1}{\underset{k=0}{\sum }}\frac{1}{S^{k+1}}\frac{1}{\left(1+\frac{\mu^{\alpha }}{S^{\alpha }}\right)}N^{k}\left(t_{0}\right).$$

Using the Taylor series expansion, we have

$$\frac{1}{\left(1+\frac{\mu^{\alpha }}{S^{\alpha }}\right)}=\overset{\infty }{\underset{n=0}{\sum }}{\left(\frac{-\mu^{\alpha }}{S^{\alpha }}\right)}^{n}.$$

Therefore,

$$L\left\{N\left(t\right)\right\}\leq \Lambda^{\alpha }\left(\frac{1}{S}-\frac{1}{S}\overset{\infty }{\underset{n=0}{\sum }}{\left(\frac{-\mu^{\alpha }}{S^{\alpha }}\right)}^{n}\right)+\overset{n-1}{\underset{k=0}{\sum }}\frac{1}{S^{k+1}}N^{k}\left(t_{0}\right)\overset{\infty }{\underset{n=0}{\sum }}{\left(\frac{-\mu^{\alpha }}{S^{\alpha }}\right)}^{n}.$$

Taking the Laplace inverse, we find

$$N\left(t\right)\leq \Upsilon^{\alpha }-\Lambda^{\alpha }\overset{\infty }{\underset{n=0}{\sum }}\frac{-{\left(\mu^{\alpha }t^{\alpha }\right)}^{n}}{\Gamma \left(\alpha n+1\right)}+\overset{n-1}{\underset{k=0}{\sum }}\overset{\infty }{\underset{n=0}{\sum }}\frac{-{\left(\mu^{\alpha }t^{\alpha }\right)}^{n}}{\Gamma \left(\alpha n+k+1\right)}t^{k}N^{k}\left(t_{0}\right).$$

Substituting the Mittag–Leffler function, we have

$$N\left(t\right)\leq \Lambda^{\alpha }\left[1-E_{1}\left(-\mu^{\alpha }t^{\alpha }\right)\right]+\overset{n-1}{\underset{k=0}{\sum }}E_{k+1}\left(-\mu^{\alpha }t^{\alpha }\right)t^{k}N^{k}\left(t_{0}\right).$$

where $E_{1}\left(-\mu^{\alpha }t^{\alpha }\right),\;E_{k+1}\left(-\mu^{\alpha }t^{\alpha }\right)$ are the series of the Mittag–Leffler function which converges for any argument, hence, we say that the solution to the model is bounded.

Thus, we define,

$$\omega =\left\{\left(S\left(t\right),\;E\left(t\right),I\left(t\right),R\left(t\right)\right)\in R_{+}^{4}:S\left(t\right),\;E\left(t\right),I\left(t\right),R\left(t\right)\leq \Lambda^{\alpha }\left[1-E_{1}\left(-\mu^{\alpha }t^{\alpha }\right)\right]+\overset{n-1}{\underset{k=0}{\sum }}E_{k+1}\left(-\mu^{\alpha }t^{\alpha }\right)t^{k}N^{k}\left(t_{0}\right)\right\}$$

hence, all solutions of (1) commencing in ω stays in ω for all t≥0.

### 4.2. Existence and Uniqueness

In this section, we study the existence and uniqueness properties of the solution of Equation (1). First, we consider the following theorem to show Lipschitz continuity.

**Theorem** **4.** *The kernels of Equation (1) satisfy the Lipschitz continuity for *$L_{i}\geq 0,\;i=1,2,\ldots ,\;4$.

**Proof.**  Let the kernel be defined as,

(3) $$g_{1}\left(t,S\right)=\Lambda^{\alpha }-\frac{\beta^{\alpha }\left(1-k^{\alpha }\right)S\left(t\right)I\left(t\right)}{f\left(I\right)}+\gamma^{\alpha }E\left(t\right)+\xi^{\alpha }I\left(t\right)-\mu^{\alpha }S\left(t\right),\;\;\;g_{2}\left(t,E\right)=p\frac{\beta^{\alpha }\left(1-k^{\alpha }\right)S\left(t\right)I\left(t\right)}{f\left(I\right)}-\left(\gamma^{\alpha }+\eta^{\alpha }+\phi^{\alpha }+\mu^{\alpha }\right)E\left(t\right),\;\;g_{3}\left(t,I\right)=\left(1-p\right)\frac{\beta^{\alpha }\left(1-k^{\alpha }\right)S\left(t\right)I\left(t\right)}{f\left(I\right)}+\eta^{\alpha }E\left(t\right)-\left(\xi^{\alpha }+\delta^{\alpha }+q^{\alpha }+\mu^{\alpha }\right)I\left(t\right),\;\;g_{4}\left(t,R\right)=\delta^{\alpha }I\left(t\right)-\mu^{\alpha }R\left(t\right).\;$$

Now,

$$\left|g_{1}\left(t,\;S\right)-g_{1}\left(t,\;S^{*}\right)\right|=\left|\left(\frac{\beta^{\alpha }\left(1-k^{\alpha }\right)I\left(t\right)}{f\left(I\right)}+\mu^{\alpha }\right)\left(S-S^{*}\right)\right|\;\leq \left(\left|\mu^{\alpha }\right|+\left|\frac{\beta^{\alpha }\left(1-k^{\alpha }\right)I\left(t\right)}{f\left(I\right)}\right|\right)\|S-S^{*}\|\;\leq \left(\left|\mu^{\alpha }\right|+\beta^{\alpha }\left(1-k^{\alpha }\right)\underset{t\in \left[0,\;\;h^{*}\right]}{\max}\left|\frac{\left(1-k^{\alpha }\right)I\left(t\right)}{f\left(I\right)}\right|\right)\|S-S^{*}\|\;\leq L_{1}\|S-S^{*}\|,\;\;\;\;\;L_{1}=\left|\mu^{\alpha }\right|+\beta^{\alpha }\left(1-k^{\alpha }\right)\underset{t\in \left[0,\;\;h^{*}\right]}{\max}\left|\frac{I\left(t\right)}{f\left(I\right)}\right|.$$

Hence,

(4) $$\left|g_{1}\left(t,\;S\right)-g_{1}\left(t,\;S^{*}\right)\right|\leq L_{1}\|S-S^{*}\|.\;$$

In a similar way, we obtain

(5) $$\left|g_{2}\left(t,\;E\right)-g_{2}\left(t,\;E^{*}\right)\right|\leq L_{2}\|E-E^{*}\|,\;\;\left|g_{3}\left(t,\;I\right)-g_{3}\left(t,\;I^{*}\right)\right|\leq L_{3}\|I-I^{*}\|,\;\;\left|g_{4}\left(t,\;R\right)-g_{4}\left(t,\;R^{*}\right)\right|\leq L_{4}\|R-R^{*}\|.$$

where

$$L_{2}=\left|\gamma^{\alpha }+\eta^{\alpha }+\phi^{\alpha }+\mu^{\alpha }\right|,\;L_{3}=\left|\xi^{\alpha }+\delta^{\alpha }+q^{\alpha }+\mu^{\alpha }\right|+\beta^{\alpha }\left(1-k^{\alpha }\right)\left(1-p\right)\underset{t\in \left[0,\;\;h^{*}\right]}{\max}\left|\frac{I\left(t\right)}{f\left(I\right)}\right|,\;$$

$\mathrm{and}\;L_{4}=\left|\mu^{\alpha }\right|.\;$□

The following Lemma converts the system to Volterra integral equations.

**Lemma** **1.** *The continuous system (1) can be transformed to equivalent Volterra integral equations*.

**Proof.**  Consider

D0CtαS(t)=g1(t, S(t)).  

On integrating fractionally, we find

(6) I0Ctα[D0CtαS(t)]=I0Ctα[g1(t, S(t))] S(t)−S(0)=1Γ(α)∫0t(t−τ)α−1g1(τ, S(τ))dtS(t)=S(0)+1Γ(α)∫0t(t−τ)α−1g1(τ, S(τ))dt.

Similarly,

(7) $$E\left(t\right)=E\left(0\right)+\frac{1}{\Gamma \left(\alpha \right)}\int_{0}^{t}{\left(t-\tau \right)}^{\alpha -1}g_{2}\left(\tau ,\;E\left(\tau \right)\right)dt,\;I\left(t\right)=I\left(0\right)+\frac{1}{\Gamma \left(\alpha \right)}\int_{0}^{t}{\left(t-\tau \right)}^{\alpha -1}g_{3}\left(\tau ,\;I\left(\tau \right)\right)dt,\;R\left(t\right)=R\left(0\right)+\frac{1}{\Gamma \left(\alpha \right)}\int_{0}^{t}{\left(t-\tau \right)}^{\alpha -1}g_{4}\left(\tau ,\;R\left(\tau \right)\right)dt.\;□$$

The following theorem provides the existence of the unique solution.

**Theorem** **5.** *Let* $0<\alpha <1,\;I=\left[0,{\;h}^{*}\right]\subseteq \mathbb{R}\;\mathrm{and}\;J=\left|S\left(t\right)-S\left(0\right)\right|\leq k_{1}.$

Let $g_{1}:I\times J\;\to \;\mathbb{R}\;$be continuous bounded function, that is ∃!M>0 such that $\left|g_{1}\left(t,\;S\right)\right|\leq M_{1}.\;$

Assume that $g_{1}$ satisfies the Lipschitz conditions. If $L_{1}k_{1}<M_{1}$, then there exist unique $S\in C\left[0,\;h^{*}\right],\;where\;h^{*}=min[h,\;\left(\frac{k_{1}\Gamma \left(\alpha +1\right)}{M_{1}}){\;}^{\frac{1}{\alpha }}\right]$ that holds the equation.

**Proof.** 
$$Let\;T=\left\{S\in C\left[0,\;h^{*}\right]:\;\;\|S\left(t\right)-S\left(0\right)\|\leq k_{1}\right\}.$$

Since T⊆ℝ and its closed set, then T is a complete metric space.

Recall that,

(8) $$\;\;S\left(t\right)=S\left(0\right)+\frac{1}{\Gamma \left(\alpha \right)}\int_{0}^{t}{\left(t-\tau \right)}^{\alpha -1}g_{1}\left(\tau ,\;S\left(\tau \right)\right)dt.\;$$

Define operator F in T by,

(9) $$F\left(S\left(t\right)\right)=S\left(0\right)+\frac{1}{\Gamma \left(\alpha \right)}\int_{0}^{t}{\left(t-\tau \right)}^{\alpha -1}g_{1}\left(\tau ,\;S\left(\tau \right)\right)dt.$$

To show that (6) satisfies Theorem 1, we have

$$\left|F\left(S\left(t\right)\right)-S\left(0\right)\right|=\left|\frac{1}{\Gamma \left(\alpha \right)}\int_{0}^{t}{\left(t-\tau \right)}^{\alpha -1}g_{1}\left(\tau ,\;S\left(\tau \right)\right)dt\right|\;\leq \frac{1}{\Gamma \left(\alpha \right)}\int_{0}^{t}{\left(t-\tau \right)}^{\alpha -1}\left|g_{1}\left(\tau ,\;S\left(\tau \right)\right)\right|dt\;\leq \frac{1}{\Gamma \left(\alpha \right)}\int_{0}^{t}{\left(t-\tau \right)}^{\alpha -1}M_{1}dt\;=\frac{M_{1}}{\Gamma \left(\alpha +1\right)}t^{\alpha }\;=\frac{M_{1}}{\Gamma \left(\alpha +1\right)}{\left(h^{*}\right)}^{\alpha }\;\leq \frac{M_{1}}{\Gamma \left(\alpha +1\right)}\frac{k_{1}\Gamma \left(\alpha +1\right)}{M_{1}}\;=k_{1}.$$

Hence,

(10) $$\left|F\left(S\left(t\right)\right)-S\left(0\right)\right|\leq k_{1}.$$

Similarly,

(11) $$\left|F\left(E\left(t\right)\right)-E\left(0\right)\right|\leq k_{2},\;\;\left|F\left(I\left(t\right)\right)-I\left(0\right)\right|\leq k_{3},\;\;\left|F\left(R\left(t\right)\right)-R\left(0\right)\right|\leq k_{4}.\;$$

Therefore F maps T onto itself.

Secondly, to show that T is a contraction, we have

$$F\left(S\right)-F\left(S^{*}\right)=S\left(0\right)-S^{*}\left(0\right)+\frac{1}{\Gamma \left(\alpha \right)}\int_{0}^{t}{\left(t-\tau \right)}^{\alpha -1}\left[g_{1}\left(\tau ,\;S\left(\tau \right)\right)-g_{1}\left(\tau ,\;S^{*}\left(\tau \right)\right)\right]d\tau .$$

Since $S\left(0\right)=S^{*}\left(0\right)$

$$\left|F\left(S\right)-F\left(S^{*}\right)\right|=\left|\frac{1}{\Gamma \left(\alpha \right)}\int_{0}^{t}{\left(t-\tau \right)}^{\alpha -1}\left[g_{1}\left(\tau ,\;S\left(\tau \right)\right)-g_{1}\left(\tau ,\;S^{*}\left(\tau \right)\right)\right]d\tau \right|\;\leq \frac{1}{\Gamma \left(\alpha \right)}\int_{0}^{t}{\left(t-\tau \right)}^{\alpha -1}\left|g_{1}\left(\tau ,\;\;S\left(\tau \right)\right)-g_{1}\left(\tau ,\;\;S^{*}\left(\tau \right)\right)\right|d\tau .\;\leq \frac{1}{\Gamma \left(\alpha \right)}\int_{0}^{t}{\left(t-\tau \right)}^{\alpha -1}L_{1}\|S-S\|d\tau \;=\frac{L_{1}}{\Gamma \left(\alpha \right)}\|S-S\|\int_{0}^{t}{\left(t-\tau \right)}^{\alpha -1}\tau^{0}d\tau \;=\frac{L_{1}}{\Gamma \left(\alpha \right)}\|S-S^{*}\|\frac{\Gamma \left(\alpha \right)}{\Gamma \left(\alpha +1\right)}t^{\alpha }\;=\frac{L_{1}}{\Gamma \left(\alpha +1\right)}\|S-S^{*}\|t^{\alpha }\;\leq \frac{L_{1}}{\Gamma \left(\alpha +1\right)}\|S-S^{*}\|{\left(h^{*}\right)}^{\alpha }\;\leq \frac{L_{1}}{\Gamma \left(\alpha +1\right)}\|S-S^{*}\|\frac{k_{1}\Gamma \left(\alpha +1\right)}{M_{1}}.$$

Hence,

(12) $$\|FS-FS^{*}\|\leq \frac{L_{1}k_{1}}{M_{1}}\|S-S^{*}\|.\;\;$$

Since, by hypothesis $\frac{L_{1}k_{1}}{M_{1}}<1$, T is contractive and has a unique fixed point.

In a similar way, we obtain

(13) $$\|F\left(E\right)-F\left(E^{*})\right\|\leq \frac{L_{2}k_{2}}{M_{2}}\|E-E^{*}\|,\;\;\|F\left(I\right)-F\left(I^{*}\right)\|\leq \frac{L_{3}k_{3}}{M_{3}}\|I-I^{*}\|,\;\;\|F\left(R\right)-F\left(R^{*}\right)\|\leq \frac{L_{4}k_{4}}{M_{4}}\|R-R^{*}\|.\;$$

Thus, Equation (1) has a unique solution. □

### 4.3. Existence of Equilibrium Solutions

Since R=N−(S+E+I), we can consider the first three equations in Equation (1) for this analysis.

Setting the first three equations in Equation (1) to zero and solving simultaneously, we find the following equilibrium solutions;

- Disease-free equilibrium $\left(E_{0}\right)$ is given as;
  $$E_{0}=\left(S^{0},0,0\right)=\left(\frac{\Lambda^{\alpha }}{\mu^{\alpha }},0,0\right).$$
- Endemic equilibrium $\left(E_{1}\right)$ is given as;
  $$E_{1}=\left(S^{1},E^{1},I^{1}\right),$$
  where
  $$S^{1}=\frac{A_{1}A_{2}f\left(I\right)}{\beta^{\alpha }\left(1-k^{\alpha }\right)\left[\left(1-p\right)A_{1}+\eta^{\alpha }p\right]},\;E^{1}=\frac{pA_{2}I^{1}}{\beta^{\alpha }\left(1-k^{\alpha }\right)\left[\left(1-p\right)A_{1}+\eta^{\alpha }p\right]},$$
  and $I^{1}$ can be obtained by solving,
  $$\Lambda^{\alpha }-\frac{\beta^{\alpha }\left(1-k^{\alpha }\right)SI}{f\left(I\right)}-\mu^{\alpha }S+\gamma^{\alpha }E+\xi^{\alpha }I=0,$$
  where
  $$A_{1}=\gamma^{\alpha }+\eta^{\alpha }+\phi^{\alpha }+\mu^{\alpha },\;and\;A_{2}=\xi^{\alpha }+\delta^{\alpha }+q^{\alpha }+\mu^{\alpha }.$$

Simplifying, we find

$$G\left(I\right)=\Lambda^{\alpha }-I\left[\frac{A_{1}A_{2}}{\left(1-p\right)A_{1}+\eta^{\alpha }p}-\left(\frac{\gamma^{\alpha }pA_{2}}{\left(1-p\right)A_{1}+\eta^{\alpha }p}+\xi^{\alpha }\right)\right]-\frac{A_{1}A_{2}f\left(I\right)}{\beta^{\alpha }\left(1-k^{\alpha }\right)\left[\left(1-p\right)A_{1}+\eta^{\alpha }p\right]}=0.$$

Since,

$$\frac{A_{1}A_{2}}{\left(1-p\right)A_{1}+\eta^{\alpha }p}-\left(\frac{\gamma^{\alpha }pA_{2}}{\left(1-p\right)A_{1}+\eta^{\alpha }p}+\xi^{\alpha }\right)>0,\;and\;f^{\prime }\geq 0,$$

then G is an increasing function.

In addition,

$$G\left(I\right)<\Lambda^{\alpha }-I\left[\frac{A_{1}A_{2}}{\left(1-p\right)A_{1}+\eta^{\alpha }p}-\left(\frac{\gamma^{\alpha }pA_{2}}{\left(1-p\right)A_{1}+\eta^{\alpha }p}+\xi^{\alpha }\right)\right],$$

then,

$$\underset{t\to \infty }{\lim}G\left(I\right)=-\infty .$$

If f(0)=1, clearly

$$G\left(0\right)=\Lambda^{\alpha }-\frac{A_{1}A_{2}}{\beta^{\alpha }\left(1-k^{\alpha }\right)\left[\left(1-p\right)A_{1}+\eta^{\alpha }p\right]}\;=\Lambda^{\alpha }\left[1-\left(\frac{\mu^{\alpha }A_{1}A_{2}}{\eta^{\alpha }p\beta^{\alpha }\left(1-k^{\alpha }\right)\Lambda^{\alpha }}+\frac{\mu^{\alpha }A_{2}}{\left(1-p\right)\beta^{\alpha }\left(1-k^{\alpha }\right)\Lambda^{\alpha }}\right)\right].$$

Thus, a unique positive solution for G exists if f G(0)>0, i.e., if

$$\frac{\mu^{\alpha }A_{1}A_{2}}{\eta^{\alpha }p\beta^{\alpha }\left(1-k^{\alpha }\right)\Lambda^{\alpha }}+\frac{\mu^{\alpha }A_{2}}{\left(1-p\right)\beta^{\alpha }\left(1-k^{\alpha }\right)\Lambda^{\alpha }}>1.$$

We shall establish,

$$\frac{\mu^{\alpha }A_{1}A_{2}}{\eta^{\alpha }p\beta^{\alpha }\left(1-k^{\alpha }\right)\Lambda^{\alpha }}+\frac{\mu^{\alpha }A_{2}}{\left(1-p\right)\beta^{\alpha }\left(1-k^{\alpha }\right)\Lambda^{\alpha }}=R_{0},$$

in the subsequent section where $R_{0}$ is the basic reproduction ratio.

### 4.4. Basic Reproduction Ratio

This is defined as the number of secondary cases caused by a single infected individual in a population of Susceptible [37]. Here we use the idea of the next-generation matrix as in [39].

Let,

Ƴ represents new infection and Ƶ represents the remaining terms in Equation (1). Then,

$$Ƴ=\left[\begin{array}{c} p\frac{\beta^{\alpha }\left(1-k^{\alpha }\right)S\left(t\right)I\left(t\right)}{f\left(I\right)}\\ \left(1-p\right)\frac{\beta^{\alpha }\left(1-k^{\alpha }\right)S\left(t\right)I\left(t\right)}{f\left(I\right)} \end{array}\right],\;\;\;and\;\;Ƶ=\left[\begin{array}{c} A_{1}E\\ -\eta^{\alpha }E+A_{2}I \end{array}\right].$$

Then,

$$Y\left(E_{0}\right)=\left[\begin{array}{cc} 0 & p\beta^{\alpha }\left(1-k^{\alpha }\right)S^{0}\\ 0 & \left(1-p\right)\beta^{\alpha }\left(1-k^{\alpha }\right)S^{0} \end{array}\right],\;\;and\;Z\left(E_{0}\right)=\left[\begin{array}{cc} A_{1} & 0\\ -\eta^{\alpha } & A_{2} \end{array}\right],$$

where $Y\left(E_{0}\right)$ is the Jacobian of Ƴ at $E_{0}$ and $Z\left(E_{0}\right)$ is the Jacobian at Ƶ at $E_{0}.$

Therefore,

$$X=Y\left(E_{0}\right)\left(Z{\left(E_{0}\right)}^{-1}\right)=\left[\begin{array}{cc} \eta^{\alpha }p\beta^{\alpha }\left(1-k^{\alpha }\right)S^{0} & A_{1}p\beta^{\alpha }\left(1-k^{\alpha }\right)S^{0}\\ \eta^{\alpha }\left(1-p\right)\beta^{\alpha }\left(1-k^{\alpha }\right)S^{0} & A_{1}\left(1-p\right)\beta^{\alpha }\left(1-k^{\alpha }\right)S^{0} \end{array}\right].$$

Note: X is the next generation matrix and the dominant eigenvalue of X is the basic reproduction ratio ($R_{0}$).

Here,

$$R_{0}=\frac{\mu^{\alpha }A_{1}A_{2}}{\eta^{\alpha }p\beta^{\alpha }\left(1-k^{\alpha }\right)\Lambda^{\alpha }}+\frac{\mu^{\alpha }A_{2}}{\left(1-p\right)\beta^{\alpha }\left(1-k^{\alpha }\right)\Lambda^{\alpha }}.$$

### 4.5. Stability Analysis of the Equilibria

In this section, we carry out a local stability analysis of both disease-free and endemic equilibrium points.

First, consider the following Jacobian matrix from Equation (1);

(14) $$J=\left[\begin{array}{ccc} -\frac{\beta^{\alpha }\left(1-k^{\alpha }\right)I}{f\left(I\right)}-\mu^{\alpha } & \gamma^{\alpha } & -\frac{\beta^{\alpha }\left(1-k^{\alpha }\right)S}{f\left(I\right)}\left(1-\frac{If^{\prime }\left(I\right)}{f\left(I\right)}\right)+\xi^{\alpha }\\ \frac{p\beta^{\alpha }\left(1-k^{\alpha }\right)I}{f\left(I\right)} & -A_{1} & \frac{p\beta^{\alpha }\left(1-k^{\alpha }\right)S}{f\left(I\right)}\left(1-\frac{If^{\prime }\left(I\right)}{f\left(I\right)}\right)\\ \frac{\left(1-p\right)\beta^{\alpha }\left(1-k^{\alpha }\right)I}{f\left(I\right)} & \eta^{\alpha } & \frac{\left(1-p\right)\beta^{\alpha }\left(1-k^{\alpha }\right)S}{f\left(I\right)}\left(1-\frac{If^{\prime }\left(I\right)}{f\left(I\right)}\right)-A_{2} \end{array}\right].$$

**Theorem** **6.** *The disease-free equilibrium (*$E_{0}$*) is locally asymptotically stable if *$R_{0}<1$.

**Proof.**  Consider Equation (14) at $E_{0},$ then we have

$$J\left(E_{0}\right)=\left[\begin{array}{ccc} -\mu^{\alpha } & \gamma^{\alpha } & \beta^{\alpha }\left(1-k^{\alpha }\right)S^{0}+\xi^{\alpha }\\ 0 & -A_{1} & p\beta^{\alpha }\left(1-k^{\alpha }\right)S^{0}\\ 0 & \eta^{\alpha } & \left(1-p\right)\beta^{\alpha }\left(1-k^{\alpha }\right)S^{0}-A_{2} \end{array}\right].$$

In echelon form, we find

(15) $$J\left(E_{0}\right)=\left[\begin{array}{ccc} -\mu^{\alpha } & \gamma^{\alpha } & \beta^{\alpha }\left(1-k^{\alpha }\right)S^{0}+\xi^{\alpha }\\ 0 & -A_{1} & p\beta^{\alpha }\left(1-k^{\alpha }\right)S^{0}\\ 0 & \eta^{\alpha } & A_{1}\left(1-p\right)\beta^{\alpha }\left(1-k^{\alpha }\right)S^{0}-A_{1}A_{2}+\eta^{\alpha }p\beta^{\alpha }\left(1-k^{\alpha }\right)S^{0} \end{array}\right].\;$$

The eigenvalues of Equation (15) are:

$$\lambda_{1}=-\mu^{\alpha }<0,\;\lambda_{2}=-A_{1},\;and\;\lambda_{3}=-A_{1}A_{2}\left(R_{0}-1\right)<0\;if\;R_{0}<1.$$

Hence, $E_{0}$ is locally asymptotically stable if $R_{0}<1.$ □

**Theorem** **7.** *The endemic equilibrium (*$E_{1}$*) is locally asymptotically stable if *$R_{0}>1$.

**Proof.**  Consider Equation (14) at $E_{1},$ then we find

$$\begin{array}{l} J\left(E_{1}\right)\\ =\left[\begin{array}{ccc} -\frac{\beta^{\alpha }\left(1-k^{\alpha }\right)I^{1}}{f\left(I\right)}-\mu^{\alpha } & \gamma^{\alpha } & -\frac{\beta^{\alpha }\left(1-k^{\alpha }\right)S^{1}}{f\left(I\right)}\left(1-\frac{I^{1}f^{\prime }\left(I\right)}{f\left(I\right)}\right)+\xi^{\alpha }\\ \frac{p\beta^{\alpha }\left(1-k^{\alpha }\right)I^{1}}{f\left(I\right)} & -A_{1} & \frac{p\beta^{\alpha }\left(1-k^{\alpha }\right)S^{1}}{f\left(I\right)}\left(1-\frac{I^{1}f^{\prime }\left(I\right)}{f\left(I\right)}\right)\\ \frac{\left(1-p\right)\beta^{\alpha }\left(1-k^{\alpha }\right)I^{1}}{f\left(I\right)} & \eta^{\alpha } & \frac{\left(1-p\right)\beta^{\alpha }\left(1-k^{\alpha }\right)S^{1}}{f\left(I\right)}\left(1-\frac{I^{1}f^{\prime }\left(I\right)}{f\left(I\right)}\right)-A_{2} \end{array}\right]. \end{array}$$

After computing the echelon form,

$$\begin{array}{cc} trace\left(J\left(E_{1}\right)\right)= & -\left(\mu^{\alpha }A_{1}+p\left(\mu^{\alpha }+\phi^{\alpha }\right)\right)\frac{\beta^{\alpha }\left(1-k^{\alpha }\right)I}{f\left(I\right)}I\left(\left(1-p\right)A_{1}+p\eta^{\alpha }\right)\\ & -\frac{\left(1-p\right)\beta^{\alpha }\left(1-k^{\alpha }\right)I}{f\left(I\right)}\left(\mu^{\alpha }+q^{\alpha }+\delta^{\alpha }\right)I\\ & -A_{2}\mu^{\alpha }\left[\left(\left(1-p\right)A_{1}+p\eta^{\alpha }\right)-\left(1-p\right)A_{1}A_{2}\left(1-\frac{If^{\prime }\left(I\right)}{f\left(I\right)}\right)\right] \end{array}$$

and

$$\begin{array}{cc} det\left(J\left(E_{1}\right)\right)= & \left(\mu^{\alpha }A_{1}+\frac{p\beta^{\alpha }\left(1-k^{\alpha }\right)\left(\mu^{\alpha }+\phi^{\alpha }\right)I}{f\left(I\right)}\right)[\left(\mu^{\alpha }+q^{\alpha }+\delta^{\alpha }\right)\left(1-p\right)\frac{\beta^{\alpha }\left(1-k^{\alpha }\right)I}{f\left(I\right)}\\ & +A_{2}\mu^{\alpha }\left(\left(1-p\right)A_{1}+p\eta^{\alpha }\right)-\mu^{\alpha }\left(1-p\right)A_{1}A_{2}\left(1-\frac{If^{\prime }\left(I\right)}{f\left(I\right)}\right)]\\ & +\left(\mu^{\alpha }\left(1-p\right)+\mu^{\alpha }\right)\left[\frac{\beta^{\alpha }\mu^{\alpha }A_{1}A_{2}\left(1-\frac{If^{\prime }\left(I\right)}{f\left(I\right)}\right)\left(1-k^{\alpha }\right)I^{1}}{\left(\left(1-p\right)A_{1}+p\eta^{\alpha }\right)+f\left(I\right)p\left(\mu^{\alpha }+q^{\alpha }+\delta^{\alpha }\right)}I\right]. \end{array}$$

Clearly, $trace\left(J\left(E_{1}\right)\right)<0$ and $det\left(J\left(E_{1}\right)\right)>0$ if $\left(\left(1-p\right)A_{1}+p\eta^{\alpha }\right)-\mu^{\alpha }\left(1-p\right)A_{1}A_{2}\left(1-\frac{If^{\prime }\left(I\right)}{f\left(I\right)}\right)>0.$ This is equivalent to $R_{0}>1.$ □

## 5. Numerical Simulations

This section is devoted to testing the performance of the proposed fractional-order model (1) under the Caputo differential operator while using a numerical explicit technique called the Adams–Bashforth–Moulton technique, also known as the fractional predictor-corrector method introduced and analyzed for its convergence and error bounds in [40,41]; s=min(1+α, 2) is the order of accuracy for the numerical technique. It is also worth mentioning that, unlike newly established numerical techniques for classical initial value problems, the present literature is not rich enough with numerical techniques for fractional order differential equations.

In this research, the parameter values used are from [28]; Λ=6, β=10, μ=0.02, γ=0.01, ξ=0.02, ϕ=0.01,q=0.02, η=0.2, δ=0.1, p∈[0,1], k=0.2, and α∈(0,1]. Consider an equi-spaced mesh $t_{i}=i\Delta t,\;i=0,1,\ldots ,\;M,\;$where M is a positive integer and $\Delta t=\frac{T}{M},$ where T is the upper limit of the closed interval of integration [0,T]. This setting leads to the following structure for the predictor part required by the numerical technique under consideration:

(16) $$S_{i+1}^{s}=S\left(0\right)+\overset{n}{\underset{i=1}{\sum }}b_{\alpha ,i,n+1}g_{1}\left(t_{i},S_{i},E_{i},I_{i},R_{i}\right),\;E_{i+1}^{s}=E\left(0\right)+\overset{n}{\underset{i=1}{\sum }}b_{\alpha ,i,n+1}g_{2}\left(t_{i},S_{i},E_{i},I_{i},R_{i}\right),\;\;I_{i+1}^{s}=I\left(0\right)+\overset{n}{\underset{i=1}{\sum }}b_{\alpha ,i,n+1}g_{3}\left(t_{i},S_{i},E_{i},I_{i},R_{i}\right),\;R_{i+1}^{s}=R\left(0\right)+\overset{n}{\underset{i=1}{\sum }}b_{\alpha ,i,n+1}g_{4}\left(t_{i},S_{i},E_{i},I_{i},R_{i}\right).$$

Similarly, for the corrector, we have

(17) $$S_{i+1}^{u}=S\left(0\right)+a_{\alpha ,n+1,n+1}g_{1}\left(t_{i},S_{i}{}^{s},E_{i}{}^{s},I_{i}{}^{s},R_{i}{}^{s}\right)+\overset{n}{\underset{i=1}{\sum }}a_{\alpha ,i,n+1}g_{1}\left(t_{i},S_{i},E_{i},I_{i},R_{i}\right),\;E_{i+1}^{u}=E\left(0\right)+a_{\alpha ,n+1,n+1}g_{2}\left(t_{i},S_{i}{}^{s},E_{i}{}^{s},I_{i}{}^{s},R_{i}{}^{s}\right)+\overset{n}{\underset{i=1}{\sum }}a_{\alpha ,i,n+1}g_{2}\left(t_{i},S_{i},E_{i},I_{i},R_{i}\right),\;\;I_{i+1}^{u}=I\left(0\right)+a_{\alpha ,n+1,n+1}g_{3}\left(t_{i},S_{i}{}^{s},E_{i}{}^{s},I_{i}{}^{s},R_{i}{}^{s}\right)+\overset{n}{\underset{i=1}{\sum }}a_{\alpha ,i,n+1}g_{3}\left(t_{i},S_{i},E_{i},I_{i},R_{i}\right),\;R_{i+1}^{u}=R\left(0\right)+a_{\alpha ,n+1,n+1}g_{4}\left(t_{i},S_{i}{}^{s},E_{i}{}^{s},I_{i}{}^{s},R_{i}{}^{s}\right)+\overset{n}{\underset{i=1}{\sum }}a_{\alpha ,i,n+1}g_{4}\left(t_{i},S_{i},E_{i},I_{i},R_{i}\right).$$

where

$$a_{\alpha ,i,n+1}=\frac{{\left(\Delta t\right)}^{\alpha }}{\Gamma \left(\alpha +2\right)},\;\mathrm{and}\;b_{\alpha ,i,n+1}=\frac{1}{\Gamma \left(\alpha +2\right)}\left[{\left(n-i+1\right)}^{\alpha }-{\left(n-i\right)}^{\alpha }\right].$$

and *s* denote the predictor and *u* represent the corrector.

Additionally,

$$\{\begin{array}{cc} n^{\alpha +1}-\left(n-\alpha \right){\left(n+1\right)}^{\alpha },\; & i=0\\ {\;(n-i+2)}^{\alpha }-2{\left(n-i+1\right)}^{\alpha +1}+{\left(n-1\right)}^{\alpha +1}, & \;1\leq i\leq n\\ 1,\; & i=n+1. \end{array}$$

**Figure 1:**
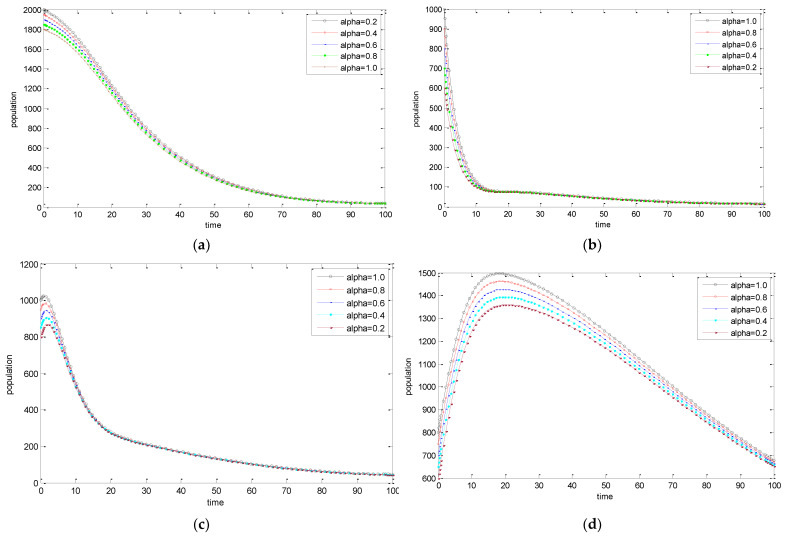
(**a**) The dynamics of the Susceptible population for varying fractional order α. (**b**) The dynamics of the Exposed population for varying fractional order α. (**c**) The dynamics of the Infected population for varying fractional order α. (**d**) The dynamics of the Recovered population for varying fractional order α.

The behavior of the Susceptible *S*(*t*), Exposed *E*(*t*), Infected *I*(*t*), and Recovered *R*(*t*) population can be seen in Figure 1a–d where the required simulations have been carried out for T = 100 days while varying the values of the fractional order parameter α. For the Susceptible population, it is observed that the population decays at a faster rate for higher values of the fractional-order parameter. On the contrary, the higher values of fractional-order parameter α lead to an increase in the Exposed, Infected, and Recovered populations.

**Figure 2:**
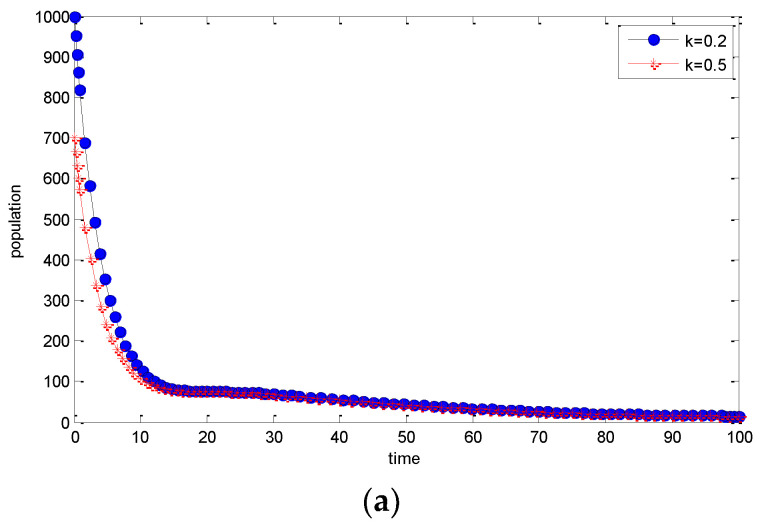

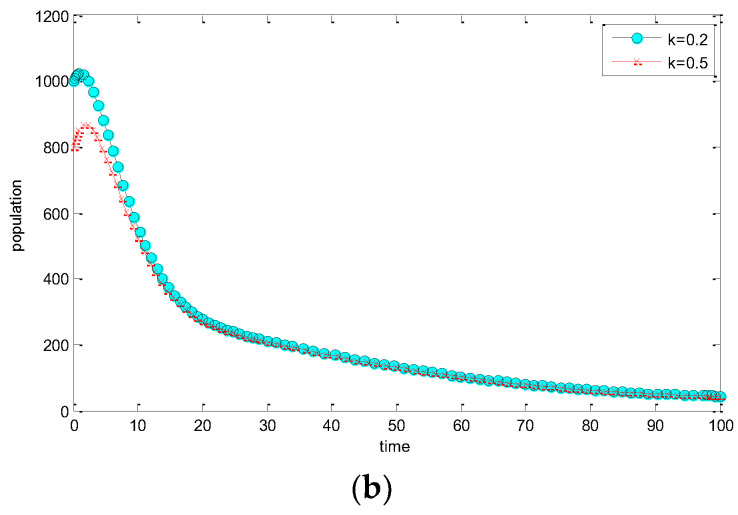
(**a**) The dynamics of the Exposed classes by increasing awareness parameter k. (**b**) The dynamics of the Infected classes by increasing awareness parameter k.

The behavior of the Exposed *E*(*t*) and Infected *I*(*t*) classes can be seen in Figure 2a,b where the required simulations have been carried out for T = 100 days while varying the values of the awareness parameter *k*. For both the Exposed and Infected classes, it can be observed that a higher value of awareness parameter k leads to a decrease in the Exposed and Infected populations.

## 6. Summary and Conclusions

In this paper, a fractional order cholera model in the Caputo sense is constructed. The transmission dynamics of the disease are studied by incorporating the saturated incidence rate into the model. Various well-posedness properties such as positivity, boundedness, existence, and uniqueness of the solution are also studied. Equilibrium solutions were computed, and their stability analysis was shown to depend on a threshold quantity, the basic reproduction ratio ($R_{0}$). It was clearly shown that if $R_{0}<1,$ the disease-free equilibrium is locally asymptotically stable, whereas if $R_{0}>1$, endemic equilibrium exists and is locally asymptotically stable. The model is found to be well-posed and more realistic than many related models in the literature due to the consideration of the saturated incidence rate.

Numerical simulations were carried out to support the analytic result and to show the significance of the fractional order from the biological point of view as well as show the significance of awareness programs in curtailing the spread of cholera in a population. From the result obtained, it is observed that the Susceptible population decay at a faster rate for higher values of the fractional-order parameter. On the contrary, the higher values of fractional-order parameter α lead to an increase in the Exposed, Infected, and Recovered populations. In addition, it is observed that for both the Exposed and Infected classes, the increase in the value of awareness parameter k leads to a decrease in the Exposed and Infected populations, respectively.

In conclusion, this paper studied a fractional order model in the Caputo sense with a saturated incidence rate. The entire well-posedness properties of the model were studied in detail. The awareness contribution in controlling cholera was studied numerically. From the findings of this research, one can see there is a need for the consideration of a saturated incidence rate in studying epidemic diseases such as cholera. Additionally, there is a need for studying well-posedness properties before making deductions on any mathematical model. From the findings of this model in particular, there is a need for relevant agencies to mount awareness programs in order to curtail the spread of cholera in a given population. Using a fractional-order to construct a mathematical model is also of paramount importance due to its hereditary properties and memory description ability. The limitation of this work is that there is a need to consider the agent-based approach of the model proposed in this paper which will help in providing more decision-making tools for the effective prevention and control of cholera and public health interventions.

## Figures and Tables

**Table 1:** Meaning of parameters.

Parameter	Meaning
*Λ*	Birthrate
β	Disease contact rate
μ	Natural death rate
ϕ	Disease-induced death rate in the Exposed class
γ	Rate at which the Exposed become Susceptible
𝜉	Rate at which the Infectious become Susceptible
𝜂	Rate at which the Exposed become Infectious
d	Disease-induced death rate in the Infectious class
δ	Rate of recovery
k	Awareness parameter
p	Fraction of individuals joining the Exposed class

## Data Availability

Not applicable.
